# Evaluate the effect of different mmps inhibitors on adhesive physical properties of dental adhesives, bond strength and mmp substarte activity

**DOI:** 10.1038/s41598-017-04340-1

**Published:** 2017-07-10

**Authors:** Pei Zheng, Hui Chen

**Affiliations:** 1 Department of Conservative Dentistry, Affiliated Hospital of Stomatology, Medical College, Zhejiang University, Hangzhou, 310006 China; 2 School of Stomatology, Zhejiang Chinese Medical University, Hangzhou, 310053 China

## Abstract

We have evaluated and compare the effect of different exogenous MMP inhibitors on adhesive physical properties of dental adhesives, bond strength, micro permeability and MMP substrate activity. 180-grit Sic paper was used to obtain the superficial dentin surface from each and every tooth after the wet grinding procedure. Dentin was exposed to four different MMP inhibitors to evaluate the effect on resin adhesive dentin interface. The four groups used in study were: 2% chlorhexidine digluconate, 2% doxycycline solution, 5% Proanthocyanidin (PR), Control Group. We evaluated and compared the four groups at each and every step of etching, bonding and resin application. Then, the immunolabeling was done with the help of the secondary antibodies with the pH of 7 and the dilution of 1:20. Amongst all the etching pretreatment groups, CHE group (Chlorhexidine etching group) revealed highest exposure to collagen fibrils than the other groups of etching. Then after the CHE group, the next group which has the second highest exposure DOE group. MMP inhibitor application for time duration of 1 minute after the etching procedures significantly improves the bond strength, exposure to collagen fibres and uniforms the dense form of dentin hybrid layer.

## Introduction

Dental adhesives are typically used for the bonding of the composite resins to the tooth structure. Dentin bonding agent’s foundation was laid by Buonocore, who in 1955 introduced etching with phosphoric acid and found that acrylic resin bonds well with etched enamel and over the years there has been evolution of dental adhesive materials^1^. Restorative dentistry is based on the adhesion between the resin based materials and the tooth substrate. The interface of the bonding between the tooth substrate and adhesive materials is called hybrid layer. Over the years, dental adhesives have been evolving, despite all the improvements in adhesive systems; the hybrid layer remains the weakest area of the adhesive dentistry.

Dentin is a complex mineralized tissue arranged in the 3-dimensional framework which is primarily of made up of 50% minerals and 30% of the organic matter and 20% volume content of the liquid by volume. The minerals in the dentin are basically, rich in the carbonate content, organic matter in the form of type I collagen fibrils and liquids in the form of plasma^2^. The authors suggested theta the dentin is difficult to bond because of its humid and organic nature^3, 4^.

The biggest limitation in the restorative dentistry is the squalor property of the adhesive dentin layer interface which further includes non-organization between the collagen and resin from the inter-fibril space^5^. The recent literature revealed that the matrix metalloproteinase (MMPs) abundant in the dentin layer after the etching process and in the saliva. MMP’s considered to be the main component of the degradation of the collagen which is present in the hybrid layer^6^. The researchers suggest that when the acidic environment has been created due to the caries, it leads to activate different MMPs, which further leads to degradation of the collagen fibrils and weakens the bond between the adhesive dentin interfaces^6^. Komori *et al*. in 2009, revealed that MMP inhibitors have a role in protection of degradation of the collagen matrix^7^.

MMPs basically are the derived from the proteolytic zinc and calcium endo-peptidases which further leads to degradation of all extracellular medium. When the dentinal MMPs are exposed and activated by the self -etching process, it leads to degradation of the type I collagen fibrils and influence the bond strength^8, 9^.

The studies have revealed that dentin hybrid layer preservation increases the dentin to restoration interface and leads to increase the bond strength. The common MMP inhibitors used are chlorhexidine, tetracycline, ammonium compounds, green tea polyphenol epigallo-catechin 3 gallate and chelating agents like ethylene dianmine acetic acid etc^10^.

Breschi *et al*. in 2010 concluded that the if chlorhexidine were placed to the etched dentin layer before the adhesive or resin placement, it will increase the bond strength and further increase the shelf life of the dental adhesive^11^. They also concluded that MMP inhibitors decrease the interfacial nano-leakage of the dental adhesive system^11^.

Tetracycline’s compounds improve the stability of dentin hybrid layer and helps in the degradation of the collagen fibrils. Doxycycline and Minocycline considered being the best MMP inhibitors in the dentin adhesive interface. The authors proved that the application of the aqueous doxycycline and minocycline after the acid etching technique increase the bond strength^12^.

Liu *et al*. in 2011 concluded that the quaternary ammonium methacrylate monomers has the inhibitory activity on MMPs and further increase the concentration of MMPs into the adhesive^13^.

Proanthocyanidins (PA) are the naturally occurring compounds that have the capability to control the MMPs activity. They act as MMP inhibitors and further control the MMP meditated diseases such as periodontitis. Many authors suggest that the PA compounds plays an important role in the inhabitation of the MMP-2,8,9^14^.

So, with this in mind, we had evaluated and compared the effects of exogenous MMPS inhibitors on adhesive physical properties of dental adhesives, the bond strength and on the substrate of mmp1 & mmp2 activity.

## Material and Methods

Before starting the research study, the permission and approval was taken from the Ethical and Research board committee of Medical College, Zhejiang University, China under the letter vide no. ZU/MC/RB/2016-72. All the methods were performed in accordance with the relevant guidelines and regulations as per the ethical and research board committee instructions.

It was the non-clinical study and no human & animals were involved in this study. The informed consent had been obtained for using the tissue sample from the subjects.

A total of 160extracted 3^rd^ Molars which were caries free and evaluated for the study. The extracted 3^rd^ molars were stored in the refrigerator at 0.c. All extracted tooth were used within one month of the post extraction period.

### Initial preparation of the specimen

The enamel surface of the tooth was aloofed and flat dentin was exposed. The roots were exposed up to the level of CEJ. Then, the diamond bur of saw shaped with the spray of water was used to remove the enamel portion. Stereomicroscope was used to evaluate the layer of enamel and its residues. Next, the dentin block surface was polished with a #600-grit wet silicon carbide (SiC) abrasive paper on a grinder (UNIPOL-820, Shenyang Kejing Equipment Manufacturing Co., Ltd) for 60 sec to standardize the smear layer.

### Method of bonding, etching and division of the groups

37% phosphoric acid was used for the acid conditioning procedure, which was done for the period of 15 seconds and further washed in the water for the same period. Dentin was further exposed to four different MMP inhibitors to evaluate the effects on the resin adhesive dentin interface.

The four groups used in study were as follows:

1. 2% chlorhexidinedigluconate
2. 2% doxycycline solution
3. 5% Proanthocyanidin (PR)
4. Control Group

A total of 160 dentin blocks which were pretreated with phosphate buffer saline at the concentration of 0.01 M with the pH of 7 were taken for the study. All the dentin blocks were equally divided into four groups, so in each group 40 dentin blocks were evaluated.

Test group I (GCHX): 2% chlorhexidine gluconate solution in the concentration of 1.5Ul was applied for 30 seconds onto the dentin blocks. The application was done by the help of micro-brush. The dentinal surface was dried with the help of an absorbent. After this, application of resin adhesive layer was done.

Test group II (GDOX): 2% of the doxycycline solution in the concentration of 1.5Ul was applied for 30 seconds onto the dentin blocks. The application was done by the help of micro brush. The dentinal surface was dried with the help of an absorbent. After this, application of resin adhesive layer was done.

Test group III: 5% Proanthocyanidin was applied the dentin blocks. The PA concentration was prepared by combining the 50% resin comonomer with 50% ethanol by the weight percentage. Proanthocyanidin was further added into the ethanol solvent at the concentration of 5.0 wt%. Then the adhesive was applied on the etched dentin and light cured further.

In the test Group IV: control group (CG), a 0.01 M phosphate buffered saline of pH 7.2 was used for etching and the dentinal surface was dried with the help of an absorbent. After this, application of resin adhesive layer was done.

Corresponding etches and the application of rinse adhesive system was done in all the groups as per the manufacturer’s instruction given (Table 1). For each group a composite resin Scotch Bond Multi Purpose, 3 M ESPE, Filtek Z250 was used for the application. In each adhesive surface, a composite resin was added in three increments and then each increment was compressed firmly. Then, the entire composite layer was light cured for 20 seconds. Light curing system with quartz halogen light was used for the photo-activation of composite resin/adhesive resin. Then the samples were rinsed in the water and further incubation was done at the room temperature for 1 day.

**Table 1:** Adhesive system, composition, and application.

Adhesive system	Compositions	Application Technique
Adper Single Bond	1. Etchant: 35% phosphoric acid	1. Apply etchant for 15 s
	2. Adhesive: Bis-GMA, HEMA, dimethacrylates, ethanol, water, photoinitiator system, methacrylate functional copolymer of polyacrylic and polyitaconic acids, 10% by weight of 5 nm-diameter spherical silica nanoparticles	2. Rinse for 15 s
		3. Blot excess water using a cotton pellet without air-drying
		4. Apply one coat of adhesive for 10 s with gentle agitation
		5. Gently air-dry (10 s at 20 cm)
		6. Apply one coat of adhesive for 10 s with gentle agitation
		7. Gently air-dry (10 s at 20 cm)
		8. Light curing for 10 s

We had evaluated and compared the four groups at each and every step of etching, bonding and resin application. So, in that way we have divided the sample into 12 further groups. These 12 groups were as follows:

#### Groups after the etching procedure

We had evaluated the four groups that were etched and examined without the adhesive or resin application, these groups were chlorhexidine etching group (CHE), doxycycline etching group (DOE), proanthocyanidin etching group (PAE) and control group.(COE)

#### Groups after the etching and adhesive application

Then we had analyzed another four groups after the etching and adhesive application, these groups were chlorhexidine adhesive group (CHA), doxycycline adhesive group (DOA),proanthocyanidin adhesive group (PAA) and control adhesive group.(COA).

#### Groups after the etching, adhesive and resin application

Then at last, we had another four groups after the application of etching, adhesive and composite resin. These groups were chlorhexidine resin group (CHR), doxycycline resin group (DOR), proanthocyanidin resin group (PAR) and control resin group. (COR)

### Effect on the MMP substrate

We had analyzed the effects of MMP inhibitors on the MMP substrates. Twenty micro liter of the adhesive with MMP inhibitor were used to coat the plates in the anaerobic environment. The plates were filled with 2% bovine serum albumin. Recombinant MMP I and II at the volume of 500 nanogram/ml were incubated overnight before the start of the procedure. In the new plate, then recombinant MMP I and II with MMP inhibitors solution was added. The procedure was done in the 96 well microtiter plates and incubation at room temperature for 24 hours. Fluorescence technique with fluorescence reader was used to evaluate the results.

### Immunolabeling specimen preparation

IgG antitype I collagen fibers and goat –IgG antibodies that were conjugated with the gold nano particles were used for the immolableling procedure. All the 12 groups were labeled with gold nanoparticles. The entire prepared sample was then washed with phosphate buffer saline at the conc. of 0.01 M and the sites of the protein were filled with the normal serum of the goat. Then the standard procedure was performed for the incubation at pH of 7.2, dilution of 1:50 at the temperature range of 4.c. The immunolabeling was done with the help of the secondary antibodies with the pH of 7 and the dilution of 1:20. The secondary antibodies were used to analyze the type I collagen. These antibodies also incubated with the entire sample by using the standard protocol. The collagen labeling was then collected. Diamond bur with continuous water spray was used to obtain the dentin blocks from the enamel – dentin junction. The blocks were pickled with 37% of the phosphoric acid. Then the debris was removed from the collagen fibrils. The sample was then washed with saline.

### Analysis of immunolabeling by field emission scanning electron microscope

The dentin blocks which are collected from the DEJ were selected from 12 groups. The sample was then fixed in the 2% gluteraldehyde solution with 0.1 M PBS solution at the pH of 7 at standard room temperature for 24 hours. The samples were then dried in the ethanol solution. The samples were further coated with gold nanoparticles using an ion-sputter coater for the time of 20 seconds. The analysis was then made under the field emission scanning electron microscope at 10 kv.

### Analysis of microtensile bond strength

International Standards Organization (ISO) Technical Specification No. 11405 has been used in this study. This specification provides guidance on substrate selection, storage, and handling as well as essential characteristics of different test methods for quality testing of the adhesive bond between restorative materials and tooth structure. It also presents some specific test methods for bond strength measurements. The shear test was carried out according to ISO/TS 11405∶2003, annex A test methods for measurement of bond strength with a shear test device as described by ISO 10477 amendment 1 and a universal testing machine (Test GmbH, Erkrath, Germany).

The groups of the samples that were resin treated i.e. CHR, DOR, PAR, COR were embedded in the plaster to avoid the cracks during the cutting process. The obtained samples were segmented into the 0.8 * 0.08 mm^2^ sticks by using the diamond bur of saw shaped and water used as coolant. The cutting was done perpendicular to the bonding surfaces. Sticks were then analyzed with help of stereomicroscope. Those sticks in which cracks appears were excluded from the sample size. 20 sticks were used for each group were taken and the incubation was done at 37 °C for 24 hour before the start of procedure. 60 sticks from the obtained different groups were stored at the room temperature and analyzed after the 3 months.

### µTBS testing

The adhesive area of each stick was measured with a micrometer, and sticks were placed on a paper towel to absorb the excess moisture. Individual sticks were bonded onto a test block of the micro-tensile testing machine (BiscoInc, Schaumburg, IL). The block was composed of two test jaws with sticks testing surfaces, linkage and slide keys. The jaws had a 2-mm gap between them and the edges were cut back at an angle to allow the testing sticks to be glued to the block in the place without the glue migrating and dripping between the jaws. We ensured that no tension was placed on the stick, and we placed a drop of glue on each jaw in the middle of the test stick mount area. We handled sticks with tweezers at the interface to place them on the jaws. All sticks were parallel to the direction of the linkage to prevent sideways vectors for tension, the stick bond interface was over the gap and no excess glue had dipped into the gap. The test block was run at a speed of 1 mm/min until the sample was broken.

Then μTBS was calculated by dividing the load at failure by the cross-sectional bonding area. The surfaces that has been fractured were sputtered with gold nanoparticles and the failure rate under the field emission scanning electron microscope were examined at the magnification of 85* at 10 kv.

The modes of the fracture were then classifies as (1) Cohesive failure in resin (CR), if the fracture occurred exclusively within the resin composite, adhesive, or both. (2) Cohesive failure in dentin (CD). (3) Interfacial failure if the fracture site was located entirely between the adhesive and dentin. (4) Mixed failure. Higher magnification was further used to evaluate the nature of fracture.

### Micro permeability Assessment

The prepared tooth with the adhesive and resin were analyzed for the micro-permeability assessment. The coronal portion of the tooth was obtained by the root removal near the CEJ. Then the whole pulp was extirpated without damaging to the layer of pre-dentine. Pulpal tissue was extirpated without altering the pre-dentin. The sample was inverted, and an aqueous solution of rhodamine B was introduced into the pulp chamber and allowed to permeate with the immersion time of 3 hrs, with the concentration of 0.5% and at 37 °C under gravity. The field emission scanning electron microscope was used to analyze the permeability of each slab with the oil immersion objective of 100 × 1.4 and excitation wavelength of 660. The rhodomine B penetration in the each and every slab was analyzed. The micro permeability assessment was done by using the modified micro permeability index.

### Ethics approval and consent to participate

Before starting the research study, the permission and approval was taken from the Ethical and Research board committee. It is a Non- Clinical Study, No Human and animal are involved in this study. The informed consent has been obtained for using the tissue sample from the subjects.

## Results

### Assessment of immunolabeling and field emission scanning electron microscopic images

**Figure 1:**
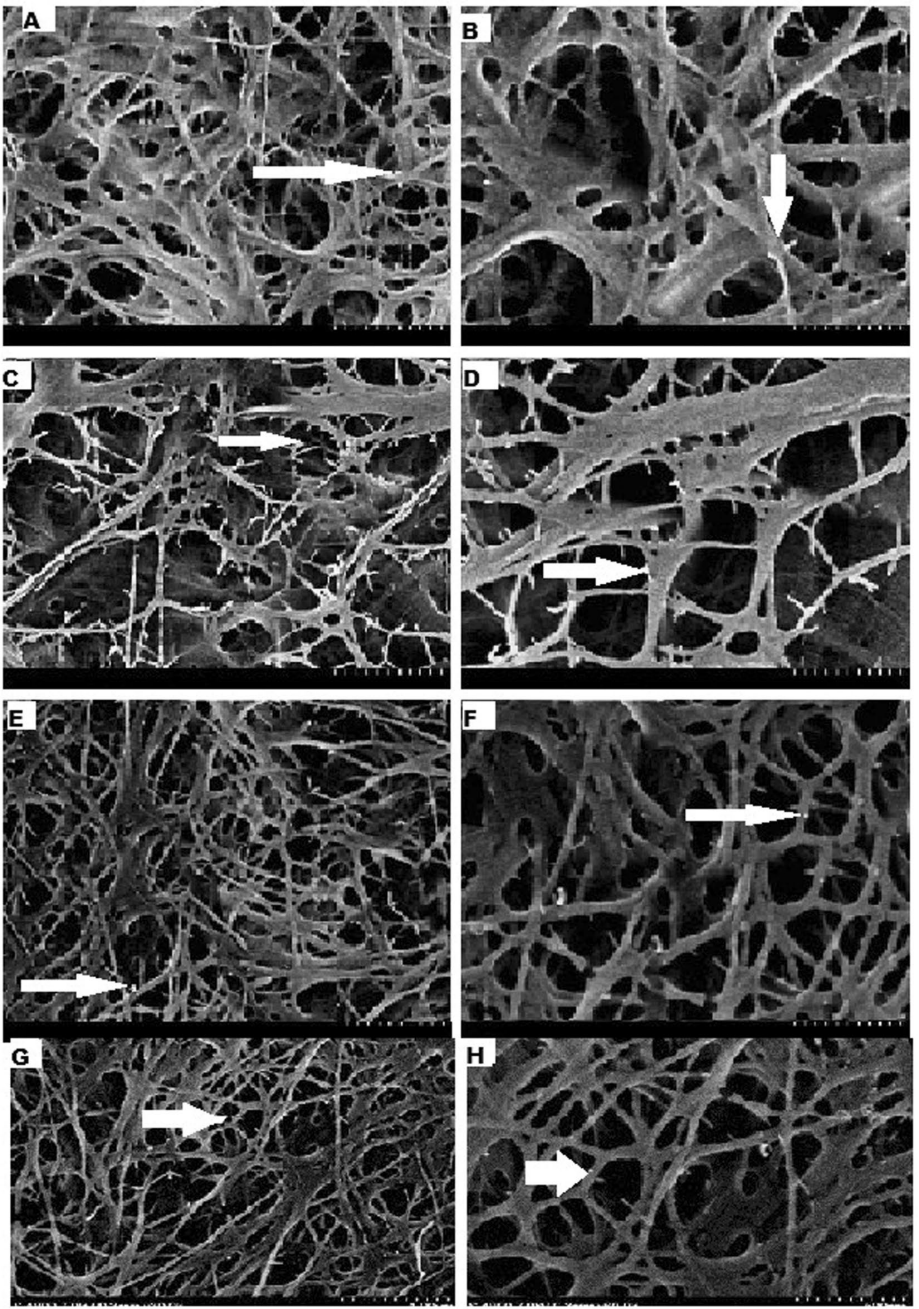
Field Emission Scanning Electron Microscopic images of the Etching groups. (**A**) COE images: Collagen fibrils are suspended in the mesh form, 3D reticular structures; gold immune labeling was not depicted without demineralization. (**B**) Amplification of COE images: 3D Collagen network more clearly seen and gold nano-particles evident. (**C**) CHE images: Visible easily depicted major form of collagen structures, Exposed collagen in the mesh form with Gold nano-particles in the secondary fibers. (**D**) Amplification of CHE images: 3D collagen fibrils clearly evident with numerous gold immunolabeling as white dots. (**E**) DOE images: Depicted of collagen structures, Exposed collagen in the mesh form with Gold nanoparticles in the secondary fibers. (**F**) Amplification of DOE images: 3D collagen fibrils clearly evident with numerous gold immunolabeling as white dots. (**G**) PAE images: Collagen fibrils are suspended in the mesh form, 3D reticular structures; Gold immunelabeling was less depicted without demineralization. (**H**) Amplification of PAE images: 3D collagen fibrils clearly evident with gold immunolabeling as white dots.

Field emission scanning electron microscopic images of the etching groups i.e CHE, DOE, PAE, and COE were first analyzed (Fig. 1A–H). The images of the demineralized part of the collagen were evident in all the images of the all the etching groups. Amongst all the etching pretreatment groups, CHE group (chlorhexidine etching group) revealed highest exposure to collagen fibrils than the other groups of etching (Fig. 1C,D). So, CHE group has dentin of maximum demineralization. Moreover, the collagen fibrils and immunolabled gold nano particles were more distinct in the secondary fibers (Fig. 1C,D). Then after the CHE group, the next group which has the second highest exposure to collagen fibers and gold labeling of nanoparticles were in DOE (doxycycline etching) group (Fig. 1E,F). The sequence of highest to lowest exposure to collagen fibers, gold nano-particles and demineralization of dentin in the etching group were as CHE > DOE > PAE > COE.

**Figure 2:**
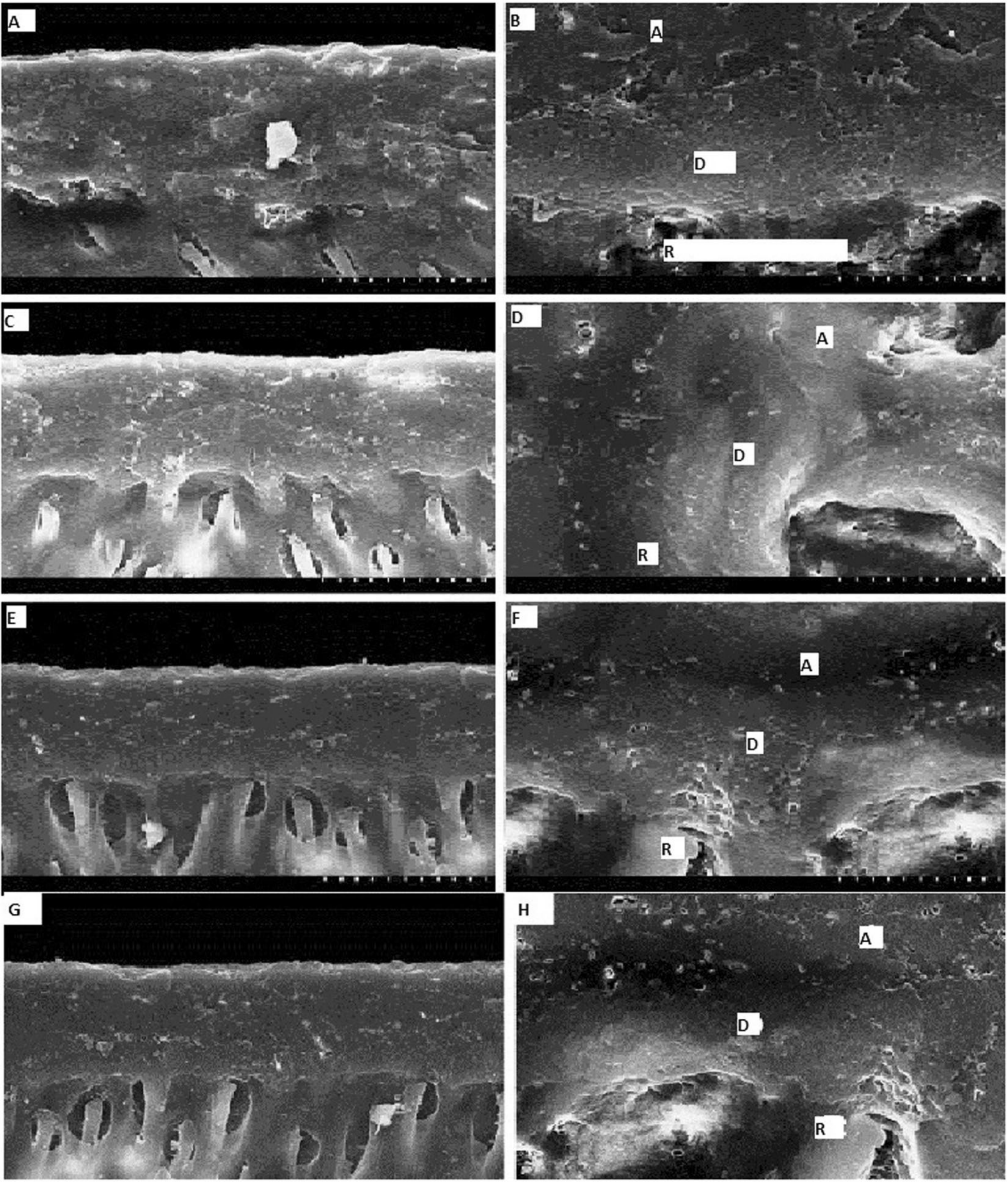
Cross Sectional Images of the Dentin Hybrid Layer after the Adhesion (R: Resin Tages, D: Dentin, A: Adhesive). (**A**) COA images: DHL layer has been depicted where exposed collagen fibers marked by gold nanoparticles. (**B**) Amplification of COA images: Exposed collagen fibers at the bottom of the DHL can be clearly seen. (**C**) CHA images: DHL is more uniform, constant and maximum dense, very high density regions are visible at the bottom of DHL where exposed collagen fibers are indicated by gold nanoparticles. (**D**) Amplification of CHA images: Exposed collagen fibers at the bottom of the DHL can be seen. (**E**) DOA images: DHL is more uniform and dense, no boundary between the bonding agent layer and the hybrid layer, high density regions are less visible at the bottom of the hybrid layer where exposed collagen fibers are indicated by gold nanoparticles. (**F**) Amplification of DOA images: Exposed collagen fibers at the bottom of the DHL can be seen. (**G**) PAA images: DHL layer has been depicted where exposed collagen fibers marked by gold nanoparticles. (**H**) Amplification of PAA images: Exposed collagen fibers at the bottom of the DHL can be clearly seen.

Then, we analyzed the different adhesive groups before the resin placement. These groups were CHA, DOA, PRA, COA (Figs. 2A–H). All the groups were had a very clear and dense dentin hybrid layer. The highest dense layer of DHL had been seen at the bottom part indicated by the heavy layer of colloidal gold particles (Figs. 2C,D). Amongst all the Adhesive groups, CHE group had the most dense DHL layer and highest exposed collagen fibrils (Figs. 2C,D). The sequence of highest to lowest dense DHL layer and exposure to collagen were as CHA > DOA > PAA > COA (Fig. 2A–H).

### Assessment of micro tensile strength and failure rate

The mean and the standard deviation for each and every group were analyzed for the micro tensile bond strength (Table 2). For the analysis and comparison between the groups regarding the micro tensile bond strength, one way ANOVA test was used. P value of less than 0.001 considers being the significant results. CHX group has the highest bond strength at 24 hours and at after the period of 3 months when compare with the DOX, PR and the control group with significant p value of less than 0.001 (Table 2).

**Table 2:** Tensile bond strength (MPa), Failure rate and Mode of failure.

Group	N	Storage Time	Mean (MPa) ± SD	F value	*P*-value	Failure Mode % (Adhesive/Cohesive/Mixed)
CHX	20	24 hours	47.91 ± 3.7	35.61	<0.001	(80.1/9.9/10)
CHX	20	3 months	42 ± 4.1	32.98	<0.001	(75.5/20.5/0.5)
DOX	20	24 hours	32.11 ± 3.2	19.90	0.07	(98.7/2.3/0)
DOX	20	3 months	30.76 ± 3.9	18.90	0.06	(90.1/9.9/0)
PA	20	24 hours	28.98 ± 3.2	16.79	0.067	(95.5/3.5/1)
PA	20	3 months	27.91 ± 3.7	17.89	0.079	(89.1/9.9/0)
Control	20	24 hours	28.11 ± 4.7	10.98	1.78	(100/0/0)
Control	20	3 months	27.89 ± 3.8	9.98	1.98	(100/0/0)

The micro-tensile bond strength of doxycycline group and PA group was higher than the control group but the results were non-significant with p value of more than 0.0001. The sequence of bond strength in our study was as follows: CHX > DOX > PA > Control group. No doubt the micro-tensile bond strength of each group after the period of 3 months was decrease but the level was not so much decreased to reveal the statistically significant results.

Fisher exact test was performed to reveal the results at the failure mode (Table 2). The analysis showed significant results. The maximum failures in each group were happened at the adhesive mode. But when the comparisons between the groups were done regarding the failure, it showed non-significant results (Table 2).

**Figure 3:**
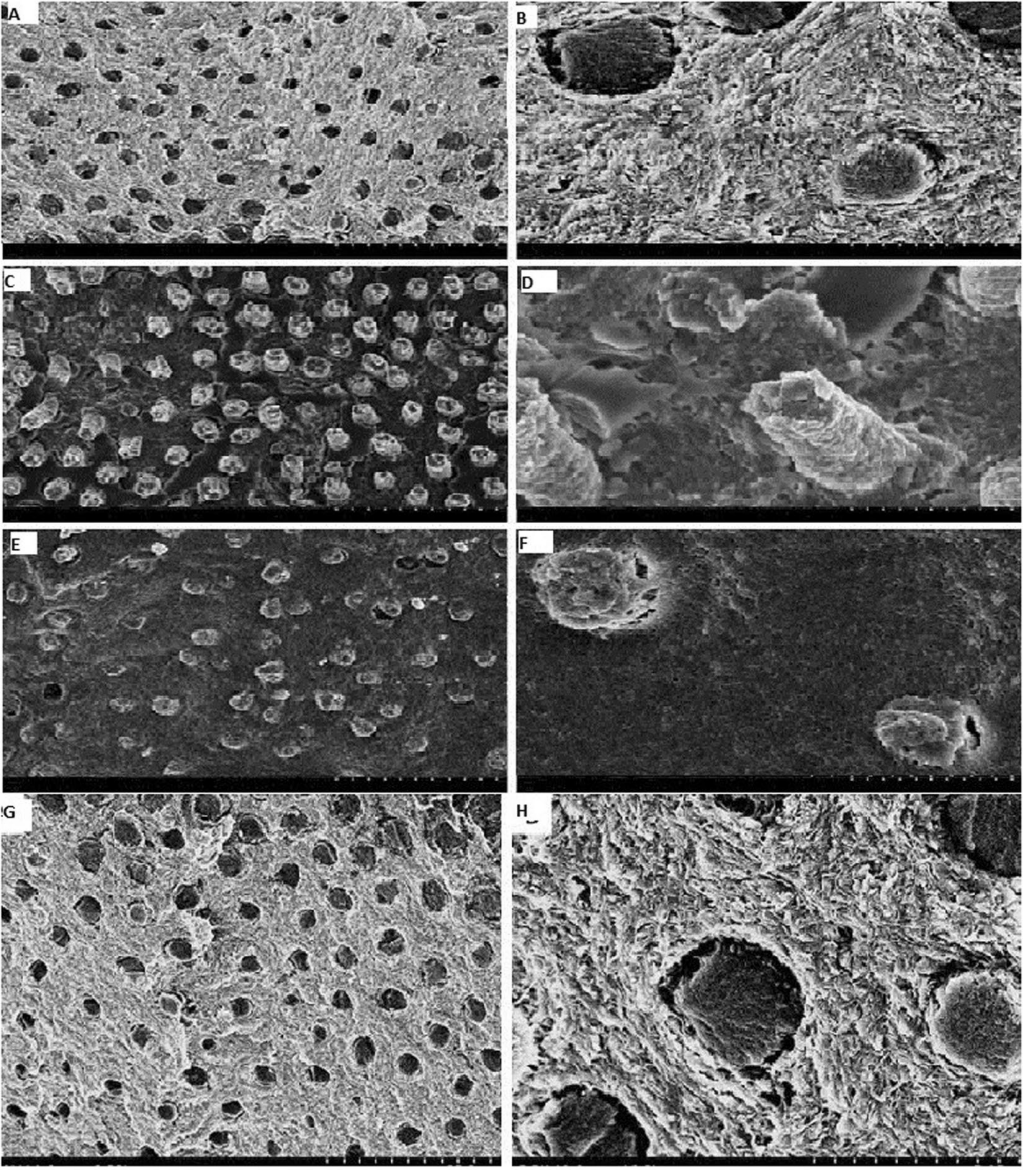
Cross Sectional Images of the after the Resin Treatment groups and Failure interfaces. (**A**) COR images: Binder adopts at the bottom surface of DHL homogeneously. (**B**) Amplification of COR images: Clear evident fracture walls between the resin and tubular wall, very short tabular walls evident with no exposure to collagen. (**C**) CHR images: Binder completely adopts the DHL and fantastic resin infiltration into the tubules of dentin. (**D**) Amplification of CHR images: Resin seems to be very much dense and much longer with no collagen exposure, no fractured walls. (**E**) DOR images: homogeneous binder coverage, most of the tubes have resin infiltration. (**F**) Amplification of DOR images: resin is thick and within tubules, with small cracks between the resin and tube wall, no exposed collagen fiber can be seen. (**G**) PAR images: Binder adopts at the bottom surface of DHL homogeneously. (**H**) Amplification of PAR images: Evident fracture walls between the resin and tubular wall, very short tabular walls evident with no exposure to collagen.

We had also analyzed the FESEM images of failed interface of the resin treatment groups after force fracture (Fig. 3A–H). CHR group shows that binder connects to the DHL completely and much better resin infiltration into dentin tubules can be seen. The resin in CHR group was obviously thicker and longer and combined with dentin tubule with no collagen fiber exposure (Fig. 3C,D). DOR group shows that there was homogeneous binder coverage and most of the tubes have resin infiltration. The resin in DOR group was thicker and within tubules, with small cracks between the resin and tube wall with no exposed collagen fiber could be seen (Fig. 3E,F). In the PAR group, we had also analyzed the homogeneous binder coverage, most of the tubes have resin infiltration and the resin was thicker and within tubules (Fig. 3G,H). In the COR group, binder covers the surface homogeneously at the bottom of the DHL and fractures between resin and tubular wall exist. The remaining tubular resin was short and no exposed collagen fibers was observed in COR group (Fig. 3A,B).

### Assessment of micro-permeability

**Figure 4:**
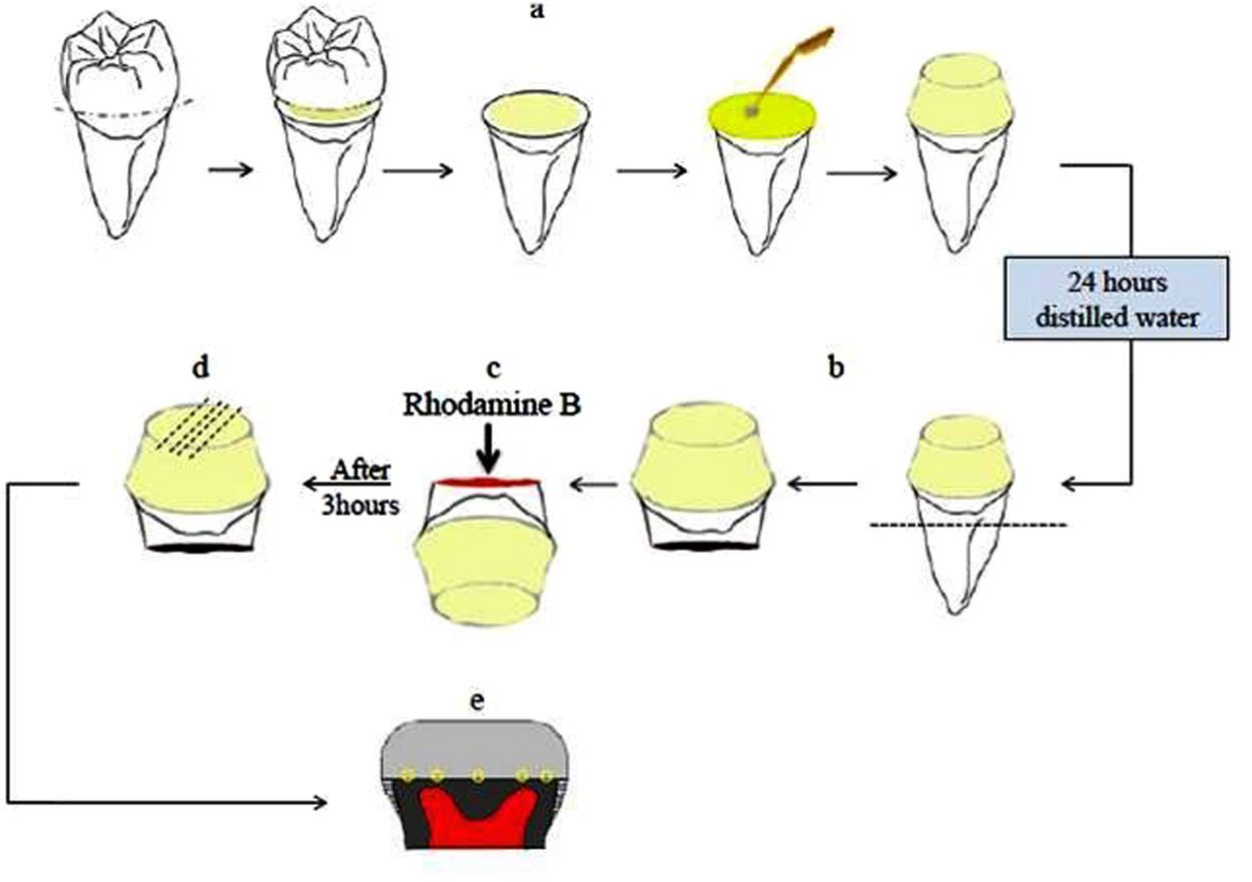
Procedure to record micro permeability.

**Figure 5:**
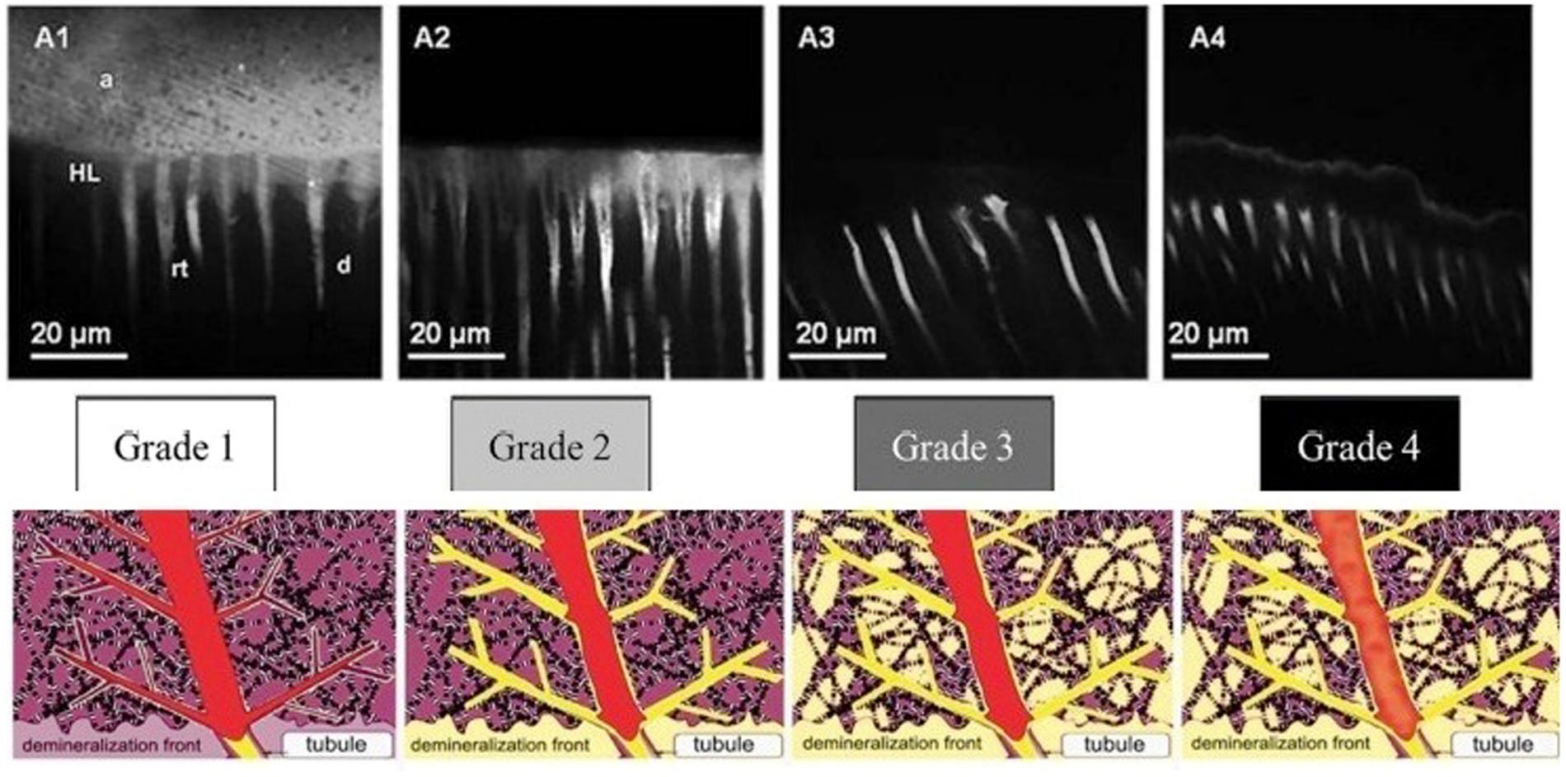
Assessment of Micro permeability: Grade 1: completely intact hybrid layer with no dye reaching it; Grade 2: the dye reaches the base of the hybrid layer; Grade 3: the dye is infiltrated within the hybrid layer completely; Grade 4: the dye is reaching the adhesive layer.

The procedure of micro-permeability assessment has been shown in the Fig. 4. The micro-permeability index used in this study is shown in Fig. 5. The grade of micro permeability is as follows; Grade 1: completely intact hybrid layer with no dye reaching it; Grade 2: the dye reaches the base of the hybrid layer; Grade 3: the dye is infiltrated within the hybrid layer completely; Grade 4: the dye is reaching the adhesive layer (Fig. 5).

The results of our study revealed that the micro-permeability in the all the groups were more or less same when comparison was made between the MMP inhibitors with the control group. The result of micro permeability shows non-significant results and further shows that MMP inhibitors had no effect on the permeability of the adhesive. CHX a strong MMP inhibitor revealed the less dye in hybrid layer when compared with the control group; however the results showed non -significant p value of 0.9 (Fig. 5).

TSM images that were captured while the rhodamine B excitation revealed the long resin threads, thick hybrid layer with the thick adhesive layer. The control group showed that there was total dye penetration into the entire thickness of the hybrid layer, while the permeability of other three MMP inhibitors group was less and detected only inside the dentinal tubules. But, comparative results showed non-significant results (Fig. 5).

### Effects of the MMP inhibitors on the substrate of MMP I and 2

All the MMP inhibitors revealed a statistically significant loss of MMP 1 and 2 activity. Analysis regarding the MMP substrate, we had evaluated the Zymographs. Zymographs revealed that the all the MMP inhibitors of the groups inhibit the MMP activity.

## Discussion

Jerome Groos and Charles have published the first report of MMPs in 1962. MMPs are basically incorporated into many physiological processes such as embryological development, wound healing, tooth eruption and many pathological processes like periodontal diseases, pulmonary emphysema, osteoporosis, malignant tumors etc^15, 16^.

MMPs has been divided into the further six groups according to their specificity, sequence similarity and domain organization. The six subgroups were 1. collagenases that included MMP-1, MMP-8, MMP-13, MMP-18: 2. stromelysins that include MMP-3 and MMP 10: 3. Gelatinases that included MMP-2, MMP-9: 4. Type IV collagenases that include MMP-7 and MMP-26: 5. Membrane type metalloproteinases that include MMP-14, MMP-15, MMP-16, MMP-17, MMP-24, MMP-25: 6. Metalloelastase that include MMP-12^17^.

The biggest challenge now a days, in the dental adhesive system is the factors that influencing the bond to dentin that include presence of amalgam restorations, caries and other tooth conditions that can affect the quality of etching and adhesion to enamel and dentin^18^. Collagen fiber quality is related to dentin hybrid layer (DHL) stability and auto-degradation of collagen fibers occurs within the DHL created by contemporary dentin bonding systems via the slow action of matrix metalloproteinases (MMPs)^18^. Hence MMP’s are implicated in degradation of poorly resin-infiltrated DHL and decrease the dentin bonding stability. Recently, with the aim of making improvements in dentin bonding stability, MMP inhibitors have been employed to pre-treat the demineralized dentin interface or have been incorporated into the bonding component to prevent activation of MMPs.

Tezvergil-MutluayA *et al*. conclude that the 37% of phosphoric acid does not dentaure the endogenous proteases. In the procedure of the acid etching of the dentin, there will be the activation of acidic environment which further promotes the MMPs that were incorporated in the peripheral layer of the dentin. This is the reason that we had used the acid etch-resin adhesive system to evaluate the effects of MMP inhibitors on the collagen network.

The researches revealed that the MMP inhibitors have effect on reducing the collagen fibers and further degradation of dentin hybrid layer^19^. But, No study till now has studied the comparative effects of different MMP inhibitors on the bond strength of adhesives and dentin hybrid layer.

Here in our study, the immunolabeling and the gold non-particles of the collagen-I and dentin hybrid layer in the different MMP Inhibitor groups were evaluated and further compared with each other and the control group. The results revealed that the CHX group at 24 hours and after the 3 months showed more constant collagen fibers and more protection of collagen fibers. In the CHX group, maximum adhesive able to penetrate the collagen network and further increase the bond of adhesiveness. This further improves the bond strength between the resin and dentin. These characteristic revealed that by preventing the activity of MMP, we can achieve more constant and dense DHL.

The next DOX group also revealed a dense layer of dentin hybrid and suggesting the good bond strength as compared to control group. The results of DOX group with the control group revealed non-significant results, but the modes of failure and fracture was more uniform. In this group fewer cracks were evaluated but, because of very longer resin tags extending into the dentin part promotes the stability of adhesiveness^20^. PA group also showed same response as shown by the DOX groups, It also proves to be good MMP inhibitor that improves further improves the bond strength.

So, in our study we favors that CHX, DOX and PA pretreatments of demineralized dentin offered better collagen networks and increased inter-fibrillar volume, which enhanced adhesive resin penetration and produced a higher quality DHL with greater immediate bonding strengths.

We had also evaluated the micro-permeability index compared with the control group. The results revealed that MMP inhibitor group has the improved rate of micro-permeability was compared with the control group. Out of this, CHX group showed maximum micro-permeability.

Successful MMP inhibitor basically has effective functional group ie. the carboxylic group and the hydroxamic group^21^. These groups have the potential to chelate the active site of MMP molecules. The Functional group provided the hydrogen bone interaction between the groups. MMP inhibitors with dentin adhesives may also increase the interlocking reaction between the MMP sub stare and the inhibitors^21^. The incorporation of MMP inhibitors with dental adhesive systems may result in the enhancement of micromechanical interlocking through the reaction between the MMP substrate and the inhibitors^22^.

Komori *et al*. evaluated the effect of 2% CHX on the long-term bond strengths of two etch-and-rinse adhesive systems to normal and caries-affected dentin and found that CHX significantly lowered the loss of bond strength after 6 months in normal dentin specimens, but did not alter the bond strength of caries-affected dentin specimens.

Kambaram M, *et al*. concluded that the application of CHX has direct effect on the normal and the carious dentin and increase the bond strength in either case even after the 12 months period^23^. In contrast to our study, many other elaborative studies revealed that the application of CHX treated healthy dentin or the carious tooth did not change the bond strength of the adhesive. Generally, the presence of carious dentin decreases bond strengths and the higher the level of caries progression, the lower the bond strengths of adhesives to carious dentin^24^.

## Conclusion

So we conclude our study by analyzing that CHX, DOX, PA group MMP inhibitors could be used for the pretreatment of dentin bonding surfaces to increase the dentin resin adhesive. MMP inhibitor application for the time duration of 1 minute after the etching procedures significantly improves the bond strength, exposure to collagen fibers and uniforms the dense form of dentin hybrid layer. MMP inhibitors also improve and avoid the degradation of DHL and collagen fibrils. The Long term effects of the different MMP inhibitors should be analyzed in the future longitudinal studies.

## Declarations

### Consent to publish

All the information provided are in original form and we are giving the consent to publish our work.

### Availability of data and materials

Not Applicable, no Personal information of any patients this is the Non Clinical Study. Other material data is included in the manuscript itself.
